# Design Strategies for Cellular Nanosponges as Medical Countermeasures

**DOI:** 10.34133/bmef.0018

**Published:** 2023-04-20

**Authors:** Shuyan Wang, Dan Wang, Mingxuan Kai, Wei-Ting Shen, Lei Sun, Weiwei Gao, Liangfang Zhang

**Affiliations:** Department of NanoEngineering, Chemical Engineering Program, and Moores Cancer Center, University of California San Diego, La Jolla, CA 92093, USA.

## Abstract

The interest in using therapeutic nanoparticles to bind with harmful molecules or pathogens and subsequently neutralize their bioactivity has grown tremendously. Among various nanomedicine platforms, cell membrane-coated nanoparticles, namely, “cellular nanosponges,” stand out for their broad-spectrum neutralization capability challenging to achieve in traditional countermeasure technologies. Such ability is attributable to their cellular function-based rather than target structure-based working principle. Integrating cellular nanosponges with various synthetic substrates further makes their applications exceptionally versatile and adaptive. This review discusses the latest cellular nanosponge technology focusing on how the structure–function relationship in different designs has led to versatile and potent medical countermeasures. Four design strategies are discussed, including harnessing native cell membrane functions for biological neutralization, functionalizing cell membrane coatings to enhance neutralization capabilities, combining cell membranes and functional cores for multimodal neutralization, and integrating cellular nanosponges with hydrogels for localized applications. Examples in each design strategy are selected, and the discussion is to highlight their structure–function relationships in complex disease settings. The review may inspire additional design strategies for cellular nanosponges and fulfill even broader medical applications.

## Introduction

Toxic molecules from various sources are major threats to public health. For example, exogenous molecules, including chemical toxicants, neurotoxins, and bacterial toxins, can cause severe injuries and even death [1–3]. Endogenous molecules, such as endotoxin, inflammatory cytokines, and pathological antibodies, are implicated in numerous diseases, including sepsis, rheumatoid arthritis, and immune hypersensitivity [4–6]. Besides toxic molecules, infectious pathogens such as bacteria and viruses also pose serious threats. For example, pathogenic bacteria secrete numerous virulence factors to disrupt host cell functions and promote colonization. Viruses target host cells for entry and exploit cellular machinery for replication [7,8]. To neutralize the threats from these molecules or pathogens, medical countermeasures, such as anti-sera, monoclonal antibodies, and small-molecule inhibitors, are commonly used [9–12]. These therapeutic platforms rely primarily on target structures for bioactivity, and the neutralization is narrow-spectrum. However, families of small-molecule toxicants or bacterial toxins are enormous, and their structures are diverse. Infectious bacteria or viruses mutate incessantly by changing their protein structures. Meanwhile, neutralizing a single or a few cytokines is inadequate to suppress the multiplex and redundant biological signaling network in complex diseases. As a result, traditional platforms often provide insufficient therapeutic efficacy [13,14]. Developing broad-spectrum medical countermeasures for more effective neutralization is critically needed.

Recently, cell membrane-coated nanoparticles, also known as “cellular nanosponges,” have attracted attention as a promising platform for medical countermeasure [15,16]. These nanoparticles are fabricated by coating natural cell membranes onto synthetic nanoparticle cores. Cellular nanosponges gain cell-like functions through surface receptors inherited from the source cells. This attribute has inspired researchers to use them for biological neutralization. The rationale is that all toxic agents, regardless of their molecular structures or modes of action, must interact with host cells for bioactivity [17]. Hence, cellular nanosponges act as cell decoys to intercept these toxic agents and block their bioactivities. The neutralization is broad-spectrum, relying on targeted cell functions rather than the target structures. It is also specific, depending on the cells chosen for the application of interest. The first formulation demonstrating this concept was red blood cell (RBC) membrane-coated poly(lactic-co-glycolic acid) (PLGA) nanoparticles (denoted “RBC-NPs”). They acted as decoys for natural RBCs, neutralizing pore-forming toxins (PFTs) secreted by bacteria [17]. Later, RBC-NPs were applied to neutralize other toxic agents, such as pathological antibodies and small-molecule toxicants, with promising results [18,19].

Following the development of RBC-NPs, various cellular nanosponges have emerged with membranes of other cell types, including platelets, white blood cells (WBCs), neuron cells, and bacteria [16,20–22]. Such diversity has widened the range of neutralizing targets for cellular nanosponges, including small-molecule toxicants, pathological antibodies, inflammatory cytokines, neurotoxins, bacteria, and viruses. As cellular nanosponges are increasingly used in complex biological systems, additional functions beyond those provided by native cell membranes become desirable. Therefore, several methods have been used to functionalize cell membrane coatings, such as lipid insertion, membrane hybridization, metabolic engineering, and genetic modification [23]. These methods lead to multitasking cellular nanosponges with enhanced neutralization capability and capacity. Meanwhile, functional cores, not only serving as substrates to stabilize the membranes but also providing neutralizing capabilities, are also used in cellular nanosponges. Some functional cores, such as oil cores, can physically absorb toxic agents [24]. Others encapsulate bioactive payloads, such as enzymes, to chemically inactivate toxic agents [25]. These designs combine the functionalities of membrane coating and cores, achieving multimodal neutralization. Furthermore, cellular nanosponges have been integrated with hydrogels to form hybrid formulations. In these formulations, nanosponges are either embedded in the hydrogel or used directly as crosslinkers to form the gel [26,27]. Nanosponge–hydrogel composite designs combine the desirable physiochemical and biological attributes of the two materials, providing effective localized neutralization against harmful agents.

**Fig. 1.**
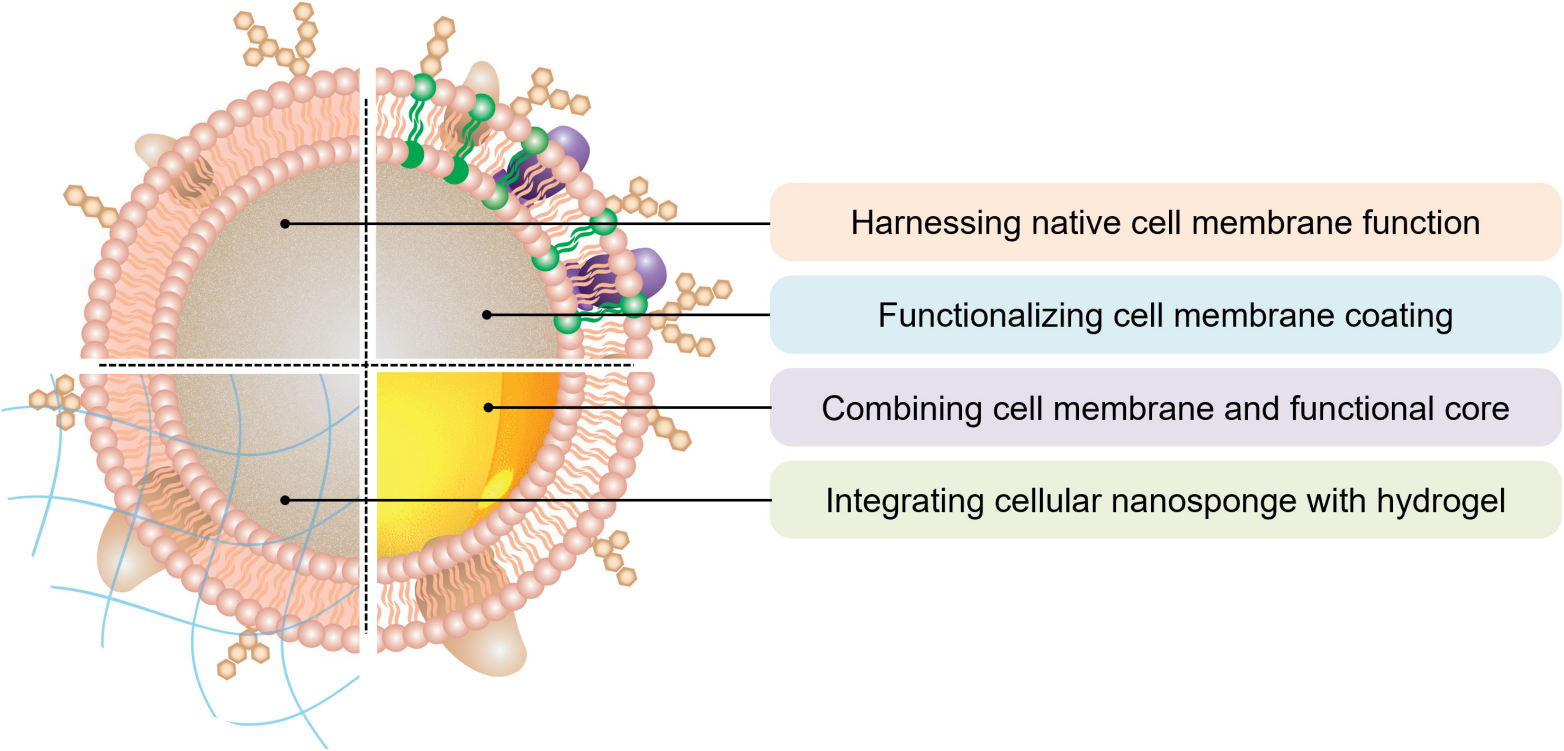
A schematic showing the major design strategies for cellular nanosponges as medical countermeasures. These designs include harnessing native cell membrane functions for biological neutralization, functionalizing cell membrane coatings to enhance neutralization capabilities, combining cell membranes and functional cores for multimodal neutralization, and integrating cellular nanosponges with hydrogels for localized applications.

This article reviews key design strategies for cellular nanosponges as medical countermeasures (Fig. 1). We highlight 4 designs that have attracted the most attention: (a) harnessing native cell membrane functions for biological neutralization, (b) functionalizing cell membrane coatings to enhance neutralization capabilities, (c) combining cell membranes and functional cores for multimodal neutralization, and (d) integrating cellular nanosponges with hydrogels for localized applications. In each design strategy, we summarize the recent advances and discuss the principles of the formulations. We emphasize their structure–function relationships and how such relationships lead to enhanced neutralization efficacy. Overall, this review demonstrates the diversity and ingenuity in cellular nanosponge designs for medical countermeasures. With continuous development, cellular nanosponges are expected to make more breakthroughs in biological neutralization.

## Harnessing native cell membrane functions for biological neutralization

The initial development of cellular nanosponges focuses on harnessing the functions of various types of cell membranes to bind with and neutralize toxins. At the same time, the synthetic cores serve primarily as a template to stabilize the membrane shell. This template also maintains nanosponge size, ensuring a high collision frequency critical for potent neutralization.

**Fig. 2.**
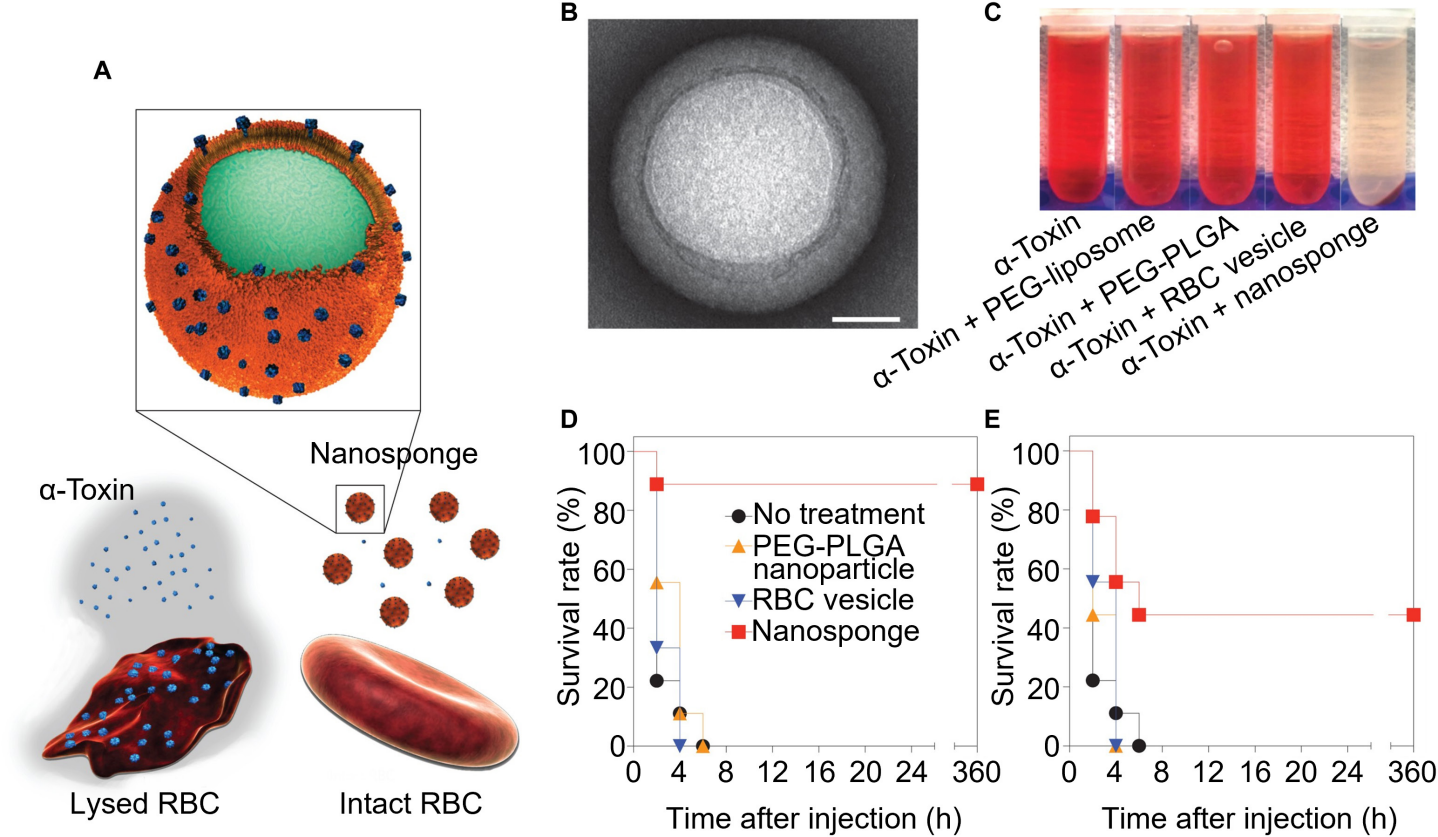
RBC nanosponges (denoted “RBC-NPs”) protect host cells from bacterial toxin attacks. (A) Schematic structure of RBC-NPs and their mechanism of neutralizing PFTs. Secreted by bacteria, PFTs perforate cell membranes and lyse the RBCs. After absorbing PFTs, RBC nanosponges divert the toxins from their cellular targets, preventing toxin-mediated hemolysis. (B) Transmission electron microscopy (TEM) visualization of a PFT-absorbed nanosponge (scale bar, 20 nm). (C) Centrifuged RBCs after incubation with α-toxin mixed in phosphate-buffered saline (PBS), pegylated PLGA nanoparticles (PEG-PLGA), pegylated liposomes (PEG-liposome), RBC membrane vesicles, or RBC nanosponges indicated the ability of the RBC nanosponges to neutralize PFTs. (D and E) Survival rates of mice over 15 d following intravenous injection of α-toxin (75 μg/kg). The RBC nanosponges, RBC vesicles, or PEG–PLGA nanoparticles were administered intravenously 2 min either before (D) or after (E) the toxin injection. All injections were performed via the tail vein (*n* = 9). Figure adapted from [17].

RBCs are long-circulating delivery vehicles in the body, enabled in part through their surface markers such as CD47 and complement regulatory proteins [28]. Based on this feature, RBC-NPs were first developed to mimic natural RBCs for prolonged circulation. However, they were soon applied to neutralize RBC susceptible toxins, especially PFTs secreted by bacteria, to perforate cell membranes for bioactivity [29]. RBC-NPs acted as host decoys, diverting toxins away from their intended cellular targets. These nanoparticles effectively neutralized alpha-hemolysin (Fig. 2) [17]. Based on the same working principle, researchers also used RBC-NPs to neutralize other PFTs, such as melittin, listeriolysin O, streptolysin O, hemotoxin, and cytolysin [30–34].

After the initial development, RBC-NPs were also applied to neutralize pathological antibodies and small-molecule toxicants. In antibody-induced hemolytic anemia (AIHA), autoantibodies attack the surface antigens on RBCs, lowering the number of RBCs in the blood [35]. Current treatments start with systemic cytotoxic drugs and B cell-depleting monoclonal antibodies, followed by splenectomy, depending on the patient’s response to the therapy. The drug regimen imposes considerable iatrogenic risk, while the spleen removal heightens susceptibility to severe infections [21]. RBC-NPs address these challenges by acting as an alternative target for autoantibodies, depleting circulating autoantibodies without contributing toxic metabolites [18]. RBC-NPs can also neutralize organophosphates (OPs), a class of small-molecule neurotoxic chemicals. These chemicals disrupt cholinergic synaptic transmissions by inactivating acetylcholinesterase (AChE), a membrane protein on neuromuscular junctions and cholinergic brain synapses, but also found on RBCs. A study showed that RBC-NPs scavenged dichlorvos (DDVP), a model OP, and improved the survival of mice challenged by a lethal dose of DDVP [19].

Besides RBC-NPs, cellular nanosponges made with membranes from other types of cells were also developed for biological neutralization. For example, platelets are a blood-circulating sentinel for vascular damage and invasive microorganisms [36]. They are also substantial in modulating innate immunity or regulating tumor growth and metastasis [37]. Platelet membrane-coated nanoparticles (denoted “PNPs”) inherit rich surface moieties of the platelet. They show platelet-like functions such as immune modulation, subendothelium adhesion, and pathogen interactions, leading to outstanding biointerfacing capability [38]. Like RBC-NPs, PNPs can neutralize anti-platelet antibodies that otherwise cause immune thrombocytopenia purpura (ITP) [39]. PNPs can also neutralize bacterial toxins, showing therapeutic potential in treating bacterial infections. For example, PNPs presented specific glycosphingolipid receptors [20]. They neutralized Shiga toxin (Stx), a toxin produced by *Escherichia coli* that causes the hemolytic uremic syndrome. In another example, PNPs absorbed methicillin-resistant *Staphylococcus aureus* (MRSA) toxins, preventing them from damaging host platelets and macrophages [40].

WBCs, such as macrophages, dendritic cells, B cells, T cells, and neutrophils, are also blood cells playing critical roles in immune modulation, pathogen clearance, and tissue repair [41]. In bacterial infection, macrophages recognize endotoxins [lipopolysaccharides (LPS)]. The subsequent immune activation triggers proinflammatory cytokine release, further potentiating macrophage activation. Like macrophages, macrophage membrane-coated nanoparticles (denoted “ΜΦ-NPs”) can also bind with LPS. Moreover, ΜΦ-NPs can neutralize inflammatory cytokines and thus suppress downstream inflammation cascades [42]. In viral infections, macrophages can interact with viruses such as severe acute respiratory syndrome coronavirus 2 (SARS-CoV-2) virus. In this regard, ΜΦ-NPs inhibited SARS-CoV-2 viral infectivity by intercepting virus binding with the host cells [43,44]. In immune disorders such as rheumatoid arthritis, neutrophils are primarily responsible for sustained joint inflammation [45]. Neutrophil membrane-coated nanoparticles (denoted “neutrophil-NPs”) sequestered immunoregulatory molecules that would otherwise stimulate endogenous neutrophils for sustained inflammation [46]. These cellular nanosponges protected joints in mouse models of experimental rheumatoid arthritis.

Like WBCs, T cells are essential immune cells recognizing diverse antigens for immune response [47]. In human immunodeficiency virus (HIV) infection, T cell depletion leads to acquired immunodeficiency syndrome (AIDS). HIV entry is initiated by the interaction between viral envelope glycoproteins, mainly gp120, and cluster of differentiation 4 (CD4) receptor, followed by binding to C–C chemokine receptor type 5 (CCR5) or C-X-C chemokine receptor type 4 (CXCR4) coreceptors on the target cells [48]. By replicating the natural adhesion of T cells toward HIV, T cell membrane-coated nanoparticles (denoted “TNPs”) neutralized HIV infectivity by diverting viral attacks away from the host cells. These nanoparticles broadly neutralized HIV-1 and suppressed viral replication through autophagy [49,50].

**Fig. 3.**
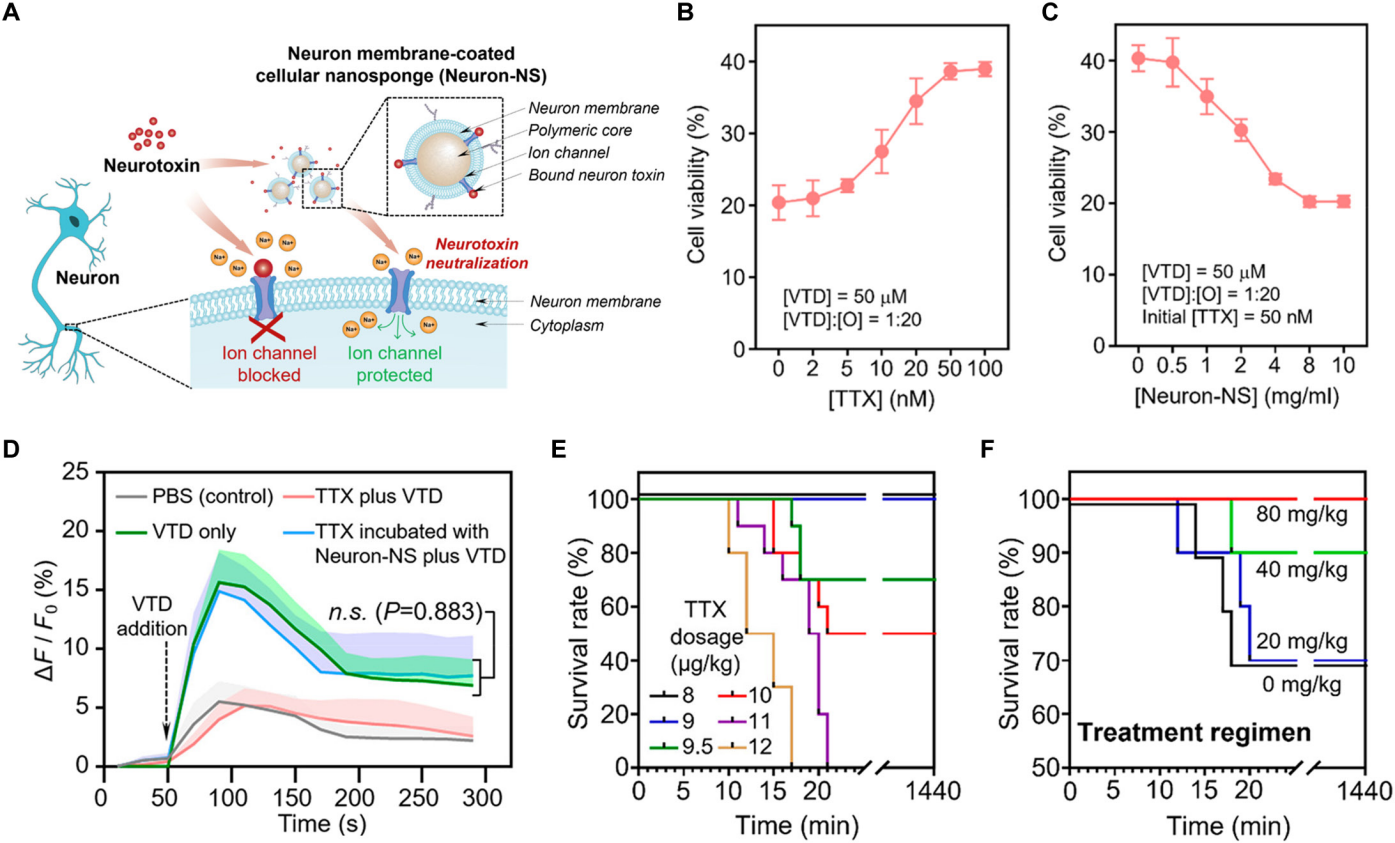
Neuronal cellular nanosponges for effective detoxification of neurotoxins. (A) Schematic of neuronal membrane-coated nanosponges (denoted “Neuron-NS”) to detoxify neuron toxins. By acting as decoys of source neurons, Neuron-NS lure neurotoxins and block them from attacking the host neuron cells. (B) Cell viability measured from Neuro-2a cells incubated with TTX in the presence of 50 μM veratridine (VTD) and 1 mM ouabain (O). TTX prevented the cytotoxicity induced by VTD and O in a dose-dependent manner. (C) Cell viability measured from Neuro-2a cells incubated with Neuron-NS-treated TTX in the presence of 50 μM VTD and 1 mM O. The initial concentration of TTX was 50 nM. Neuron-NS neutralized TTX, resulting in a dose-dependent decrease in cell viability. (D) Time-lapse fluorescence change of Neuro-2a cells as an indication of Ca^2+^ influx. Cells were exposed to PBS (control), VTD only, TTX and VTD, and TTX incubated with Neuron-NS and VTD. *F*_0_ is the mean fluorescence intensity of the cells without VTD. Δ*F* is the difference between the fluorescence intensity at given time points and *F*_0_. (E) Survival rates of mice over 24 h after the subcutaneous injection of TTX. (F) Survival rates of mice over 24 h in a treatment regimen. Neuron-NS were administered intravenously 1 min after a single subcutaneous injection of 9.5 μg/kg TTX. Data presented as mean ± SD. In all data sets, *n* = 3 independent experiments using the same batch of Neuron-NS. Figure adapted from [21].

Recently, neuronal membrane-coated nanosponges (denoted “Neuron-NS”) were developed for neuron toxin neutralization. Once neurotoxins attack the neurons, nervous system functions will be impaired, leading to serious health problems [51,52]. For example, tetrodotoxin (TTX) binds with voltage-dependent sodium channels on the neuron surface, disrupting the channel functions. Neuron-NS acted as the decoy of the source neurons. As an alternative target, they bound with TTX, blocking them from attacking the host neuron cells (Fig. 3) [21]. In a mouse model of TTX intoxication, neuron-NS effectively protected mice challenged by a lethal dosage of TTX.

Besides mammalian cells, bacterial membranes are also attractive coating materials. Pathogenic bacteria adhere to the host cells via surface receptors for host colonization. For example, *Helicobacter pylori* bacteria have species-specific tissue tropism due to many adhesins on their surface, leading to gastritis ulcers and gastric cancer [53]. Polymeric nanoparticles coated with *H. pylori* outer membrane (denoted “OM-NPs”) competed with the bacteria to occupy binding sites on the host cells, effectively reducing bacterial attachment. Such competition also dissociated colonized *H. pylori* from infected mouse stomach tissue [22].

These examples demonstrate how a basic core-shell design of cellular nanosponges can leverage cell membrane functions for detoxification, making them a powerful nanomedicine platform. Cellular nanosponges have opened detoxification opportunities for critical diseases currently challenging or untreatable. Their roles will become even more important as the understanding of the cellular mechanisms of diseases deepens.

## Functionalizing cell membrane coatings to enhance neutralization capabilities

The desire to increase the versatility and capabilities of cell membrane-coated nanoparticles for complex detoxification applications has grown tremendously [54,55]. One approach is introducing additional functions onto the cell membranes and using the function-enhanced membranes for nanoparticle coating. Typical functionalization methods include lipid insertion, membrane hybridization, genetic modification, and metabolic engineering.

Lipid insertion was first developed to integrate lipid-linked functional ligands onto the cell membranes subsequently used for coating [56]. Recently, lipophilic molecules such as PFTs or hydrophobic compounds were inserted into the cell membrane for compelling nanosponge designs. In one study, melittin was inserted into the RBC membrane together with oleyl-oxyethyl-phosphoryl-choline (OOPC), a hydrophobic phospholipase A2 (PLA2) inhibitor, and the membrane was coated onto PLGA cores for enhanced PLA2 inhibition [57]. In this design, melittin acts as a PLA2 attractant, working with the membrane lipids to “lure” PLA2. Meanwhile, OOPC “kills” PLA2 upon enzymatic attack. By integrating melittin and OOPC within the cell membrane, this cellular nanosponge design (denoted “L&K-NP”) avoids toxicity associated with free melittin and OOPC molecules. The study found that these nanosponges were nonhemolytic and showed no cytotoxicity. In contrast, an equivalent amount of free OOPC or melittin elicited dose-dependent hemolysis or cell death. L&K-NP protected mice challenged by a lethal dose of venomous PLA2.

**Fig. 4.**
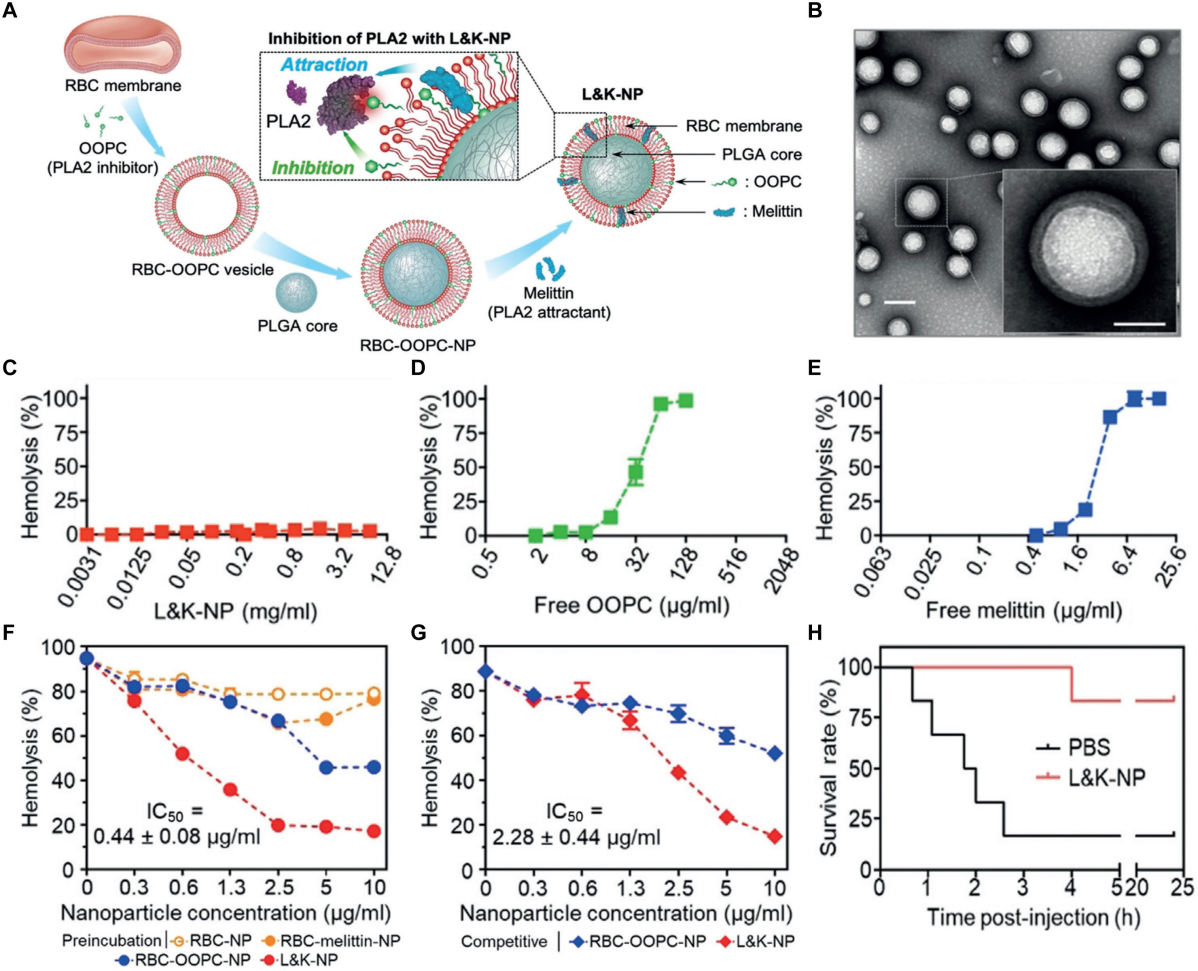
A cellular nanosponge to “lure and kill” PLA2. (A) Schematic illustration of the synthesis of L&K-NPs and its application to lure and kill PLA2. (B) Representative TEM images of L&K-NPs. Samples were stained with uranyl acetate (scale bar, 100 nm). Insert: a zoomed-in image of an L&K-NP (scale bar, 50 nm). (C) Hemolytic activity of L&K-NPs (*n* = 3). (D) Hemolytic activity of free OOPC (*n* = 3). (E) Hemolytic activity of free melittin (*n* = 3). (F) Hemolytic activity of PLA2 preincubated with RBC-NPs, RBC-melittin-NPs, RBC-OOPC-NPs, or L&K-NPs at various nanoparticle concentrations. Hemolysis was evaluated at 60 min after mixing PLA2 with RBCs (*n* = 3). (G) Hemolytic activity of PLA2 mixed simultaneously with RBCs and RBC-OOPC-NPs or L&K-NPs at various concentrations. Hemolysis was evaluated at 60 min after mixing PLA2 with RBCs (*n* = 3). (H) Survival rates of mice over 24 h following an injection of PLA2 (1.0 mg/kg), followed immediately by an injection of L&K-NPs (2.5 mg/kg). All injections were performed intravenously via the tail vein (*n* = 6). Figure adapted from [57].

Based on a similar design, researchers developed another L&K-NP formulation to treat severe acute pancreatitis [58]. This time, the nanosponges were synthesized first by mixing macrophage membrane and MJ-33, a hydrophobic PLA2 inhibitor, followed by coating the membrane onto PLGA cores. The coated nanosponges were then mixed with melittin for insertion, resulting in the final formulation (denoted “MΦ-NP(L&K)”). The macrophage membrane is a natural target that lures the PLA2 molecule. The inserted melittin attracts PLA2 to react with the nanosponge membrane. Once the PLA2 degrades the membrane, MJ-33 is released, which inhibits the PLA2. In the study, MΦ-NP(L&K) neutralized PLA2 activity in the sera of mouse and human patients with acute pancreatitis. They also suppressed PLA2-induced inflammatory response. In mouse models of mild and severe acute pancreatitis, MΦ-NP(L&K) effectively reduced disease-associated inflammation, tissue damage, and lethality (Fig. 4).

Membrane hybridization is another functionalization method where membranes of different cell types are mixed and hybridized for nanoparticle coating. Hybridization is often achieved through mechanical forces such as shearing, extrusion, and sonication. Nanoparticles coated with hybrid cell membranes inherit the advantage of each parent cell type and enable multifaceted biological functions. This strategy was first tested with hybridizing RBC membrane and platelet membrane to form hybrid membrane-coated PLGA nanoparticles (denoted “[RBC-P]NPs”) [59]. Like platelets, these nanoparticles targeted cancer cells and atherosclerosis. Like RBCs, they presented AChE and neutralized DDVP. Later, a similar hybrid membrane was prepared and coated onto a fuel-free nanorobot (denoted “RBC-PL-robot”) for neutralizing bacterial toxins and bacteria [60]. Relying on acoustic propulsion, RBC-PL-robots moved like natural motile cells and avoided biofouling. The movement enhanced the binding and detoxification efficiency. These nanorobots neutralized PFTs targeting the RBC membrane component and removed bacteria with affinity to the platelet membrane component. Using several model PFTs and MRSA USA300, researchers demonstrated rapid toxin neutralization and bacterial removal by RBC-PL-robots.

Genetic modification is another popular design strategy to gain new functions for cell membrane coating. It allows for the sustainable and stable presentation of desired functional ligands compared to other methods [61,62]. For example, MEDI4893, a neutralizing monoclonal antibody against MRSA α-toxin, was genetically fused onto HEK 293T cell surface, and the modified cell membrane was coated onto meso-tetrakis (4-sulfonatophenyl) porphyrin (TPPS) sonosensitizer to treat MRSA infection [63]. The fused antibodies allowed the nanosponges to inhibit α-toxin more effectively than the unmodified counterpart. The antibody also targeted nanosponges to the infection site. Combined with ultrasound-enabled bacterial killing, this nanosponge eradicated MRSA bacteria in a mouse model of systemic MRSA infection. In another study, umbilical vein endothelial cell (UVEC) membrane was coated onto PLGA nanoparticles to treat experimental rheumatoid arthritis [64]. Due to the rich receptors on the UVEC membrane, these cellular nanosponges neutralized inflammatory cytokines and protected the inflamed joints. Researchers further engineered this membrane to overexpress tumor necrosis factor-related apoptosis-inducing ligand (TRAIL), which targeted the joint macrophage. Hydroxychloroquine, a first-line antirheumatic drug, was also loaded into the PLGA core for targeted delivery. When intravenously injected into collagen-induced arthritic mice, these nanosponges accumulated more in the joints than unmodified nanosponges. They effectively suppressed joint inflammation and decreased the disease severity.

**Fig. 5.**
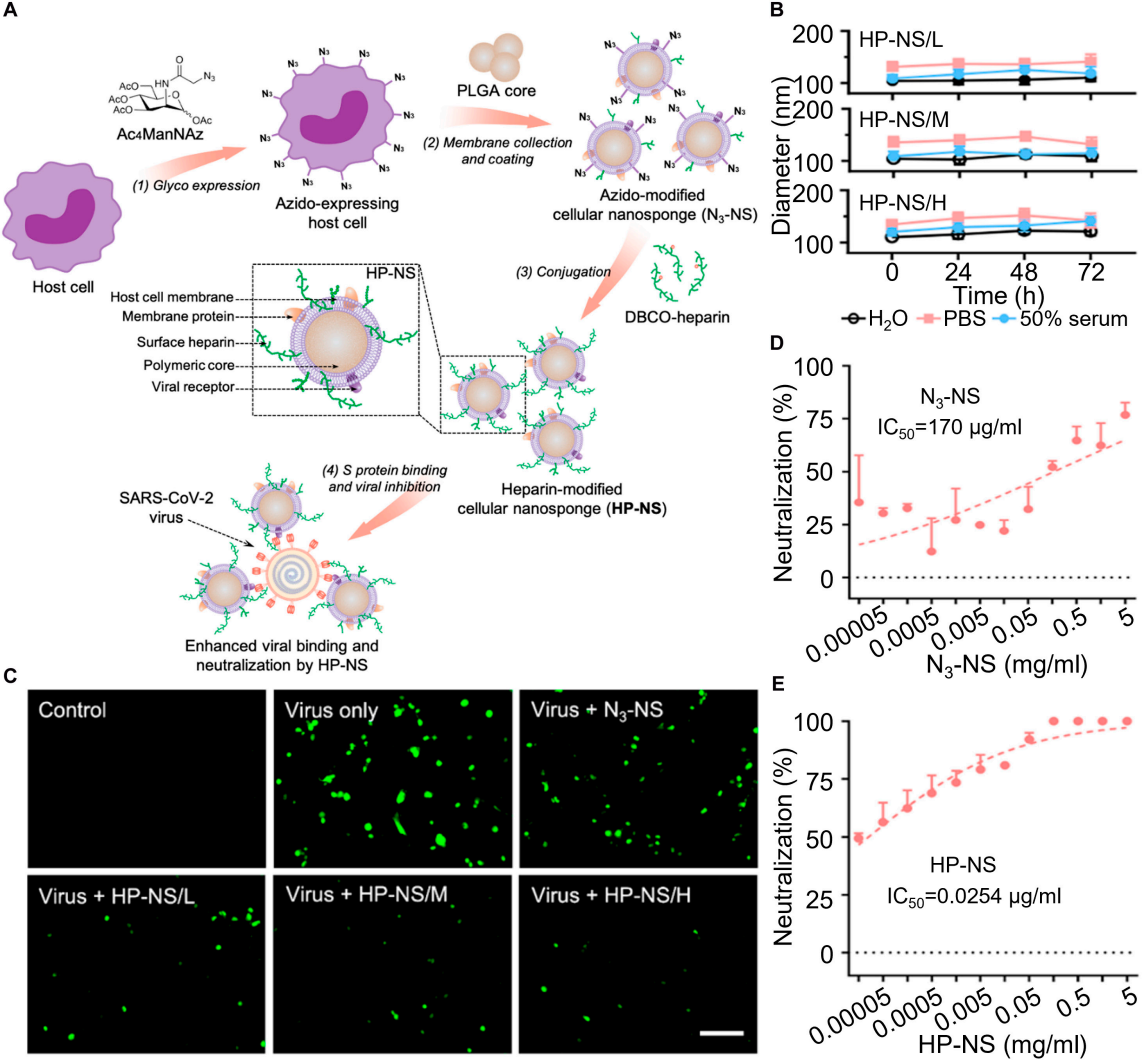
Surface glycan modification of cellular nanosponges to promote SARS-CoV-2 inhibition. (A) Schematic illustration of engineering surface glycans onto cellular nanosponges to promote SARS-CoV-2 inhibition. The host cells are first incubated with *N*-azidoacetylmannosamine-tetraacylated (Ac_4_ManNAz) to express azido groups. Their membranes are collected and coated onto polymeric nanoparticle cores made of PLGA to form cellular nanosponges expressing azido groups (denoted “N_3_-NS”). Then, heparin functionalized with the DBSO groups (DBCO-heparin) is conjugated to azido-NS through copper-free click chemistry, forming heparin-modified cellular nanosponges (denoted “HP-NS”). The HP-NS are then examined for binding ability with SARS-CoV-2 S proteins and inhibition efficacy against the viral infectivity. (B) Hydrodynamic size of HP-NS/L, HP-NS/M, and HP-NS/H in H_2_O, 1× PBS, and 50% serum over 72 h (*n* = 3). (C) Representative fluorescence images of NL-20 cells without or with HP-NS (25 μg/ml) under the infection of SARS-CoV-2 pseudovirus (5 × 10^7^ viral genes per well) for 24 h. Green represents the fluorescent proteins in the nuclei (scale bars, 100 μm). (D) The neutralization against live SARS-CoV-2 infection by HP-NS was tested on Vero E6 cells (*n* = 3). (E) The neutralization against live SARS-CoV-2 infection by HP-NS/H was tested on Vero E6 cells (*n* = 3). Data are presented as mean + SD. Horizontal dashed lines mark the zero levels. IC_50_ (half-maximal inhibitory concentration) values were derived from the variable slope model using GraphPad Prism 8. Figure adapted from [44].

Recently, researchers also used natural cellular metabolic pathways to modify cell membranes toward effective biological neutralization. Metabolic engineering manipulates natural biosynthetic pathways of a cell to anchor functional moieties onto the cell surface, which bestows cellular nanosponge with desirable functionalities [65]. For example, researchers used this method to modify macrophage membrane to neutralize SARS-CoV-2 viruses [44]. In the study, the membrane expressed azide group (N_3_) through metabolic engineering and was coated onto PLGA cores to form N3-functionalized nanoparticles. These nanoparticles were then conjugated with dibenzocyclooctyne (DBSO)-modified heparins via copper-free click reaction, forming heparin-modified cellular nanosponges (denoted “HP-NS”). Notably, the heparin density on the nanosponge surface can be controlled through N3 expression density. Compared to unmodified macrophage nanosponges, HP-NS showed a higher binding capacity with SARS-CoV-2 S proteins and higher neutralization potency against live SARS-CoV-2 viruses. Such neutralization was dose-dependent. It also depended on the heparin coverage; a higher level of heparin coverage resulted in a higher neutralization efficiency (Fig. 5).

Overall, these examples demonstrate that functionalizing cell membranes is a powerful design strategy to enhance the multifunctional and multitasking capability of cellular nanosponges. As rich cell membrane properties and cellular functions are continually discovered, this method is expected to bring new opportunities for broader neutralization applications.

## Combining cell membranes and the functional cores for multimodal neutralization

Using functional cores with biological neutralization capabilities in cellular nanosponge design has recently attracted much attention. The cell membrane coating works together with the functional cores in such constructs to achieve multimodal neutralization capabilities.

In some designs, the cores physically absorb toxicants, contributing to the overall neutralization efficacy. For example, a biomimetic nanoabsorbent (denoted “NAb”) was engineered by coating the RBC membrane onto PLGA cores to neutralize doxorubicin (DOX) [66]. Both NAb and PLGA cores showed high DOX absorbing capability due to electrostatic attraction between positively charged DOX molecules and negatively charged PLGA cores. When NAb and DOX were incubated with B16-F10 melanoma cells, NAb significantly reduced DOX-related cell damage and protected cell viability. Another study used RBC membrane-coated oil nanosponges (denoted “Oil-NS”) to neutralize OPs [24]. In this design, the oil core nonspecifically absorbed OPs through hydrophobic interactions, and the RBC membrane specifically bound OPs through their AChE receptor. Oil-NS absorbed more OPs than oil nanodroplets or RBC-PLGA nanoparticles alone. When treating mice injected subcutaneously with a lethal dose of paraoxon (POX), Oil-NS relieved the poisoning symptoms and increased the mouse survival rate to 100% after intravenous administration. RBC-NPs at the exact dosage showed no survival benefits.

**Fig. 6.**
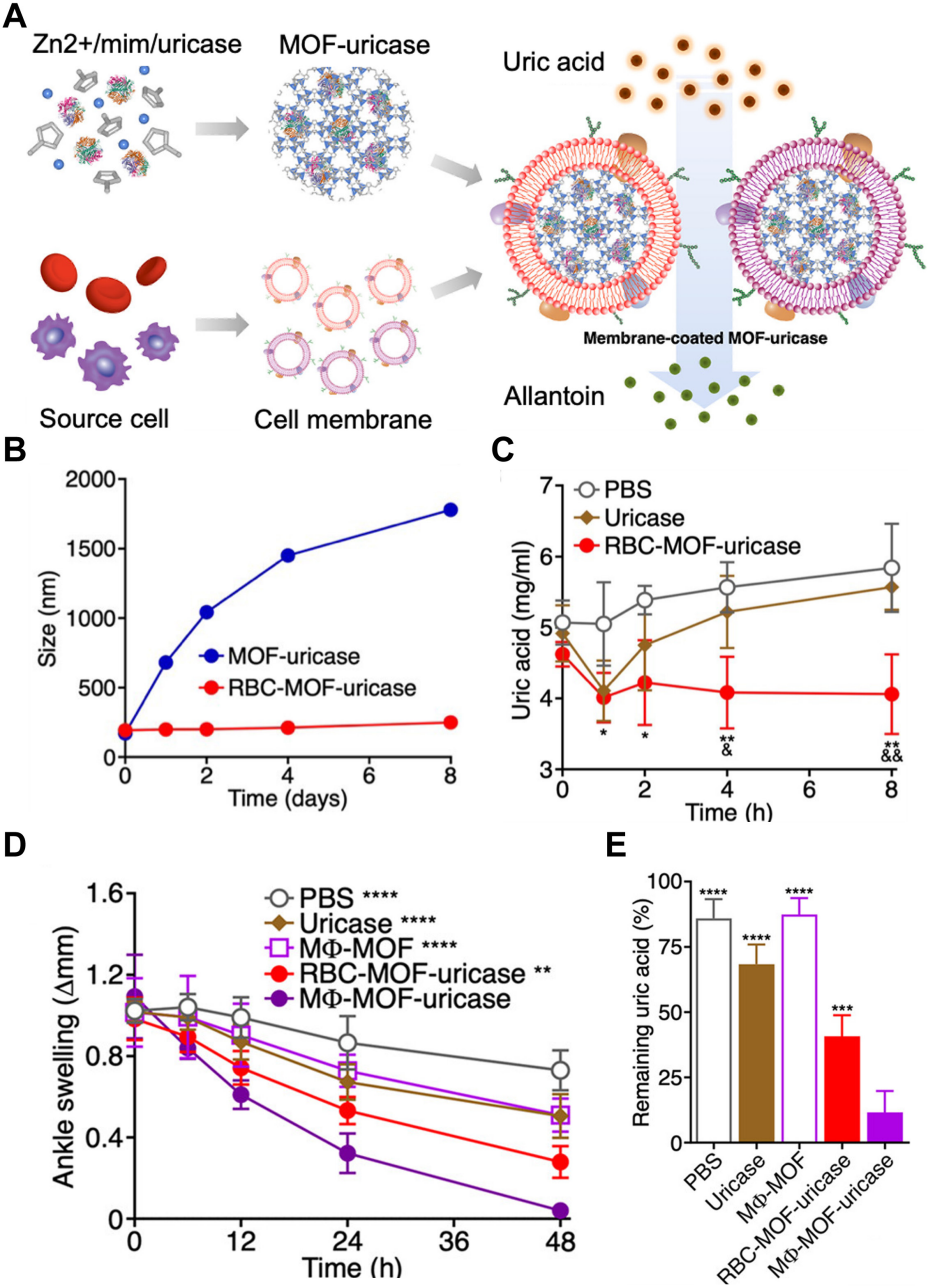
Cell membrane-coated uricase-loaded MOF nanoparticles for hyperuricemia suppression and gout treatment. (A) Schematic drawing of cell membrane-coated uricase-loaded MOF nanoparticles and their enzymatic activity in degrading uric acid. (B) Colloidal stability of RBC-MOF-uricase and bare MOF-uricase over 8 d in PBS (*n* = 3, mean ± SD). (C) Time-series serum uric acid levels of hyperuricemic mice after being intravenously injected with PBS, free uricase, or RBC-MOF-uricase (*n* = 4, mean ± SD). [PBS versus RBC-MOF-uricase difference: *P < 0.05, **P < 0.01; free uricase versus RBC-MOF-uricase difference: ^&^*P* < 0.05, ^&&^*P* < 0.01; one-way analysis of variance (ANOVA) test]. (D) Time-series ankle swelling size of mice with gout after being intra-articularly injected with PBS, free uricase, MΦ-MOF, RBC-MOF-uricase, or MΦ-MOF-uricase (*n* = 4, mean ± SD). (Difference compared with MΦ-MOF-uricase: **P < 0.01, ****P < 0.0001; one-way ANOVA test.) (E) Remaining uric acid level in the ankle swelling of the gout mice 48 h after intra-articular treatment with PBS, free uricase, MΦ-MOF, RBC-MOF-uricase, or MΦ-MOF-uricase (*n* = 4, mean ± SD). (Difference compared with MΦ-MOF-uricase: **P < 0.01, ****P < 0.0001; one-way ANOVA test.) Figure adapted from [25].

In some nanosponge designs, the cores contain toxin-degrading enzymes that work with the membrane for neutralization. Cell membrane-coated metal–organic framework (MOF) nanoparticles (denoted “CM-MOF”) stand out as the MOF cores allow for de novo and sequential encapsulation of different enzymes with high loading capacity. Some CM-MOF degrade the toxicants that directly participate in enzymatic reactions. For example, RBC membrane-coated MOF-uricase nanoparticles (denoted “RBC-MOF-uricase”) neutralized serum uric acid to treat hyperuricemia (Fig. 6) [25]. The RBC membrane coating stabilized the MOF nanoparticles that would otherwise aggregate in the biological buffers. In a murine model of hyperuricemia, RBC-MOF-uricase rapidly reduced serum uric acid levels and kept them at the baseline for 8 h. In contrast, free uricase only transiently decreased serum uric acid levels, which rebounded 2 h after the injection. Similarly, macrophage membrane-coated MOF-uricase nanoparticles (denoted “MΦ-MOF-uricase”) also showed a high uric acid-degrading activity [25]. Besides decreasing systemic serum uric acid levels, MΦ-MOF-uricase neutralized proinflammatory cytokines and treated localized joint inflammation caused by insoluble uric acid deposits. In a murine gout model, free uricase, RBC-MOF-uricase, and MΦ-MOF-uricase were injected intra-articularly. MΦ-MOF-uricase outperformed other formulations in reducing gout severity.

Some CM-MOF-NPs decompose metabolic waste or degrade the toxins not directly through the enzymatic reaction but by reactive oxygen species (ROS) generated from the enzymatic reactions. In a recent study, neutrophil membrane-coated MOF nanoparticles encapsulating glucose oxidase and chloroperoxidase (denoted “GCZM”) were developed to neutralize *S. aureus* in its infection [67]. In this design, the neutrophil membrane allowed the nanoparticles to target the disease sites, similar to neutrophil recruitment in bacterial infections. Glucose oxidase and chloroperoxidase cooperated to catalyze ROS cascade reactions, producing hypochlorous acid (HClO) from glucose. The highly oxidative HClO molecules denatured bacterial proteins and nucleic acids for bacterial killing. When intravenously injected into *S. aureus*-infected mice, GCZM circulated longer than its uncoated counterpart. GCZM decreased *S. aureus* density in infected tissues and shrunk the bacterium-induced skin lesion within 5 d after injection.

The initial success of CM-MOF motivated further study to explore how cell membrane dynamics affect the enzymatic reaction. For example, RBC or macrophage membrane-coated and organophosphorus hydrolase (OPH)-loaded MOF nanoparticles (denoted “RBC-MOF-OPH” or “MΦ-MOF-OPH”) were tested for POX detoxification [16]. Depleting cell membrane cholesterol improved the enzymatic activity of both nanosponges. The improvement correlated with the cholesterol level decrease and membrane permeability increase. By decreasing cholesterol content from 100% to 30%, OPH enzymatic activity increased more than 70% for both RBC-MOF-OPH and MΦ-MOF-OPH, making these nanosponges more effective in neutralizing POX. The study suggests that modulating the membrane cholesterol level can fine-tune the neutralization activity of CM-MOF.

Antibacterial inorganic cores are also attractive for designing cellular nanosponges. These cores contribute physical rather than biochemical mechanisms against the bacteria with a low risk of eliciting resistance [68]. For example, gold–silver nanocages (denoted “GSNCs”) were used for their photothermal antibacterial activity under near-infrared (NIR) laser irradiation [69]. In this design, macrophage membrane-coated GSNCs bound to bacteria because of the bacterium-targeting receptors on the nanoparticle surface. Researchers pretreated macrophage cells with *S. aureus* to boost receptor levels, and the potency of nanosponges coated with the membrane of these cells increased. The final formulation (denoted “SA-M-GSNC”) was injected intravenously into mice with *S. aureus*-infected skin. Under NIR irradiation, SA-M-GSNC inhibited bacterial growth and prevented skin lesion development effectively.

**Fig. 7.**
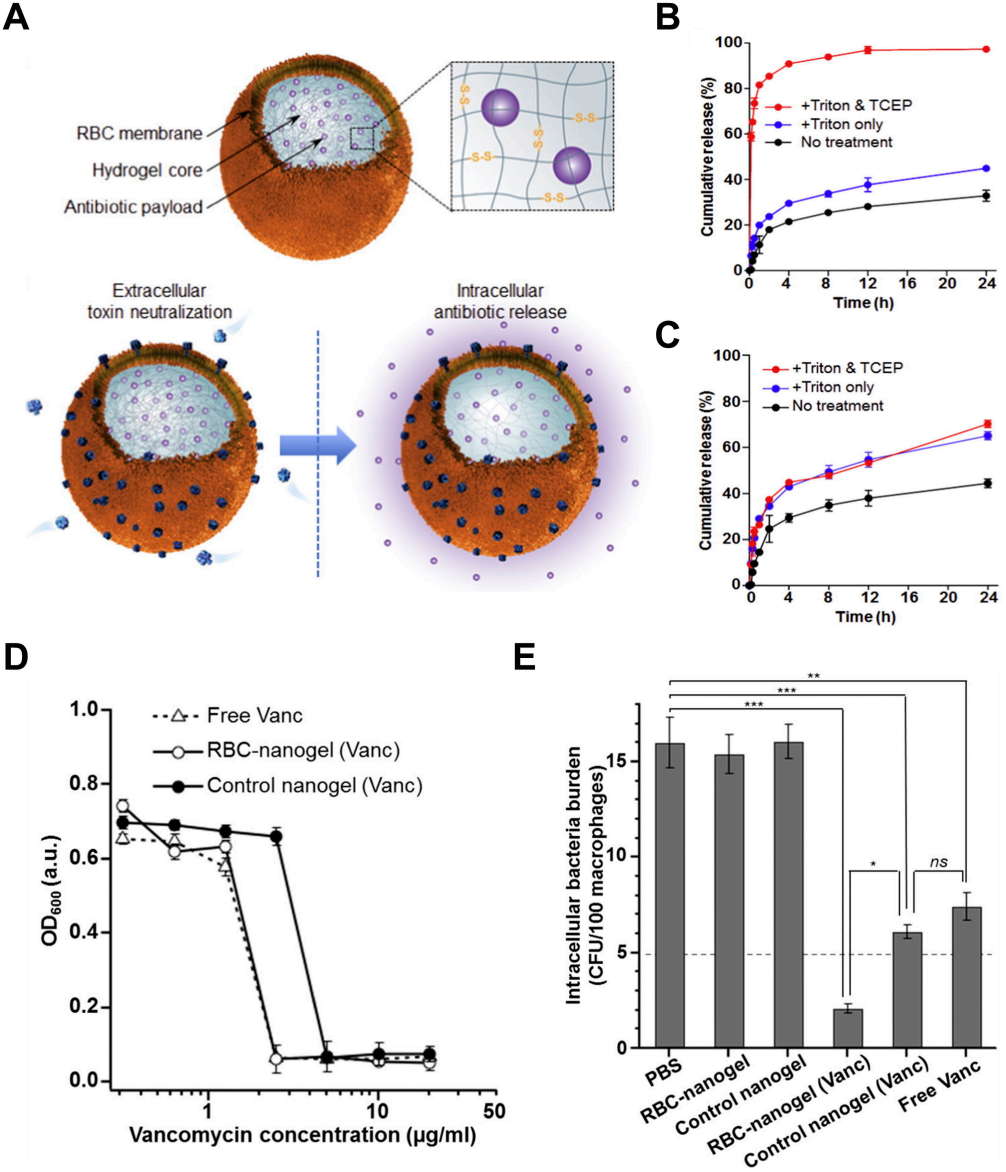
RBC-nanogels for combinatorial neutralization of MRSA and MRSA-generated toxins. (A) Schematic drawing of RBC-nanogels for extracellular MRSA toxin neutralization and intracellular redox-responsive antibiotic release to directly kill MRSA bacteria. (B) The 24-h cumulative release profile of vancomycin from redox-responsive RBC-nanogels after treatment with PBS, Triton X-100, or Triton X-100 followed by tris(2-carboxyethyl)phosphine (TCEP). Triton X-100 was adopted to simulate endosome degradation conditions. TCEP can initiate redox reactions in the microenvironment. (C) The 24-h cumulative release profile of vancomycin from nonresponsive control RBC-nanogels after treatment with PBS, Triton X-100, or Triton X-100 followed by TCEP. (D) OD_600_ (optical density at 600 nm wavelength) of MRSA USA300 bacterial suspension incubated with free vancomycin, vancomycin-loaded RBC-nanogels, and vancomycin-loaded control nanogels at 37 °C for 24 h. Triton X-100 and TCEP were added to all groups to simulate the intracellular environment. (E) Intracellular bacteria burden of MRSA USA300-infected THP-1 macrophages after incubation with PBS, empty RBC-nanogels, control nanogels, vancomycin-loaded RBC-nanogels, vancomycin-loaded control-nanogels, and free vancomycin. The bacterial burden was measured by disrupting the macrophages, collecting the intracellular bacteria, incubating them on a trypticase soy agar plate, and quantifying the number of colonies. Student’s *t* test: ns, not significant, *P < 0.5, **P < 0.01, ***P < 0.001. Figure adapted from [70].

Some nanosponges are designed to inhibit intracellular bacteria. The bacterial killing relies on traditional antibiotics loaded in the core but released in response to the intracellular reducing environment. For example, antibiotic-loaded and RBC membrane-coated hydrogel nanoparticles (denoted “RBC-nanogels”) were made to treat intracellular MRSA infection (Fig. 7) [70]. Cystine dimethacrylate, a redox-responsive crosslinker, was used to prepare the nanogel cores in this design. These cores were loaded with vancomycin and coated with RBC membrane. This RBC-nanogel neutralized bacterial toxins outside the cells, making the bacteria more prone to immune clearance. They released the vancomycin inside the cells to kill the MRSA bacteria. Compared to free antibiotics and nonresponsive RBC-nanogel controls, RBC-nanogels responded to the intracellular reducing potential and released antibiotics rapidly. Besides, due to effective PFT neutralization by the RBC membrane coating, the bacterial virulence decreased, and their uptake by THP-1 macrophages increased. This effect contributed significantly to bacterial inhibition.

In summary, the above examples depict cellular nanosponges with functional cores as powerful platforms for medical countermeasures. Applying oil and MOF cores in designing these nanoparticles broadens the selection of neutralization targets. Integration of enzymes compatible with MOF cores makes these nanoparticles more versatile. The core has also inspired the incorporation of a novel mechanism for synergistic and more dynamic bacterial neutralization. In future development, novel nanoparticle cores with intriguing functions can be combined with the cell membrane coating, leading to more effective biological neutralization.

## Integrating cellular nanosponges with hydrogels for localized applications

The advantages of cellular nanosponges also inspire hybrid formulations that integrate the nanosponges with hydrogels for local detoxification. Such nanosponge–hydrogel composite judiciously integrates two materials into one robust hybrid system with unique physicochemical and biological properties that either building block cannot achieve independently.

**Fig. 8.**
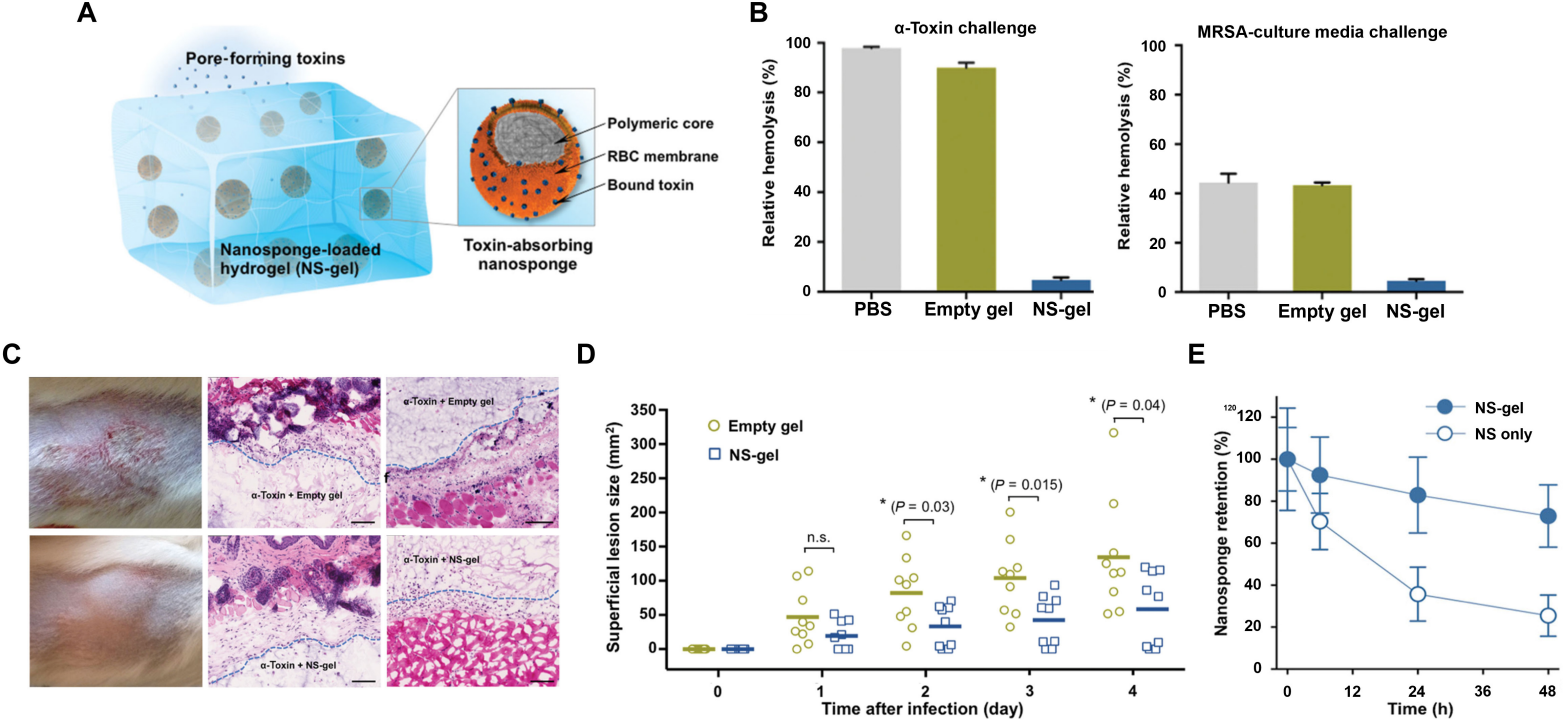
Hydrogel retaining toxin-absorbing nanosponges for local treatment of MRSA infection. (A) Schematic illustration of a nanosponge-loaded hydrogel (NS-gel) hybrid system applied for the antivirulence treatment of local infection induced by MRSA. (B) In vitro toxin neutralization. The hemolysis of centrifuge-collected RBCs was measured after respectively incubating RBCs with PBS, empty gel, or NS-gel premixed with either α-toxin or MRSA-culturing medium. (C) In vivo toxin neutralization. Mice were subcutaneously injected with α-toxin, followed by the treatment of either empty gel or NS-gel in the same route. After 72 h of toxin injection, the developed skin lesions were photographed (left). The hematoxylin and eosin (H&E)-stained histological sections showed biological phenomena like edema, apoptosis, necrosis, and immune infiltration in the epidermis (middle), and muscular damage implied by interfibril edema, tears on muscle fibers, and neutrophil extravasation from surrounding vasculature (right). (D) In vivo antibacterial efficacy against MRSA infection. NS-gel or empty gel premixed with MRSA 252 in 1 × 10^9^ CFU (colony-forming unit) was subcutaneously injected into the backs of mice (*n* = 9). Skin lesion sizes were then monitored on days 1 to 4 after injection. The bars represent median values. *P < 0.05, n.s*.:* not significant. (E) In vivo nanosponge retention by the hydrogel. The nanosponge retention was measured within 48 h of injection (*n* = 3). Error bars represent the SD; ***P < 0.001. Figure adapted from [71].

In one study, RBC-membrane-coated PLGA nanosponges (denoted “RBC-NS”) were integrated into an acrylamide-based hydrogel for treating MRSA infection (Fig. 8) [71]. In this case, nanosponges were first fabricated and mixed with acrylamide monomer and poly(ethylene glycol) dimethacrylate (PEGDMA) crosslinker for gelation. Optimizing crosslinker concentration ensured that the nanosponge-embedded hydrogel (denoted “NS-gel”) possessed a lower viscosity without nanosponge leakage. In vitro, the NS-gel absorbed and neutralized MRSA-produced α-toxin as effectively as free RBC-NS. In vivo, the hydrogel network retained encapsulated RBC-NS at the local injection site of mice. Compared to 80% leakage within 2 h of subcutaneously injected free RBC-NS, only 20% loss of nanosponges was found with NS-gel. The outstanding nanosponge retention promoted the subcutaneously administered NS-gel to neutralize α-toxins and effectively prevented skin lesion formation. When challenged with locally injected MRSA bacteria, the NS-gel-treated mice showed smaller superficial lesion sizes than those injected with empty gel, revealing the potential of the NS-gel system for treating local bacterial infection.

After NS-gel development, technology like 3-dimensional bioprinting was explored to synthesize NS-gel with the advantages of high speed and adjustable hydrogel shape [72]. In one study, RBC-NS were premixed with poly(ethylene glycol) diacrylate (PEGDA) monomers, followed by ultraviolet-irradiated photopolymerization. The NS-gel showed little RBC-NS leakage and effectively absorbed toxins at least 3-fold more than the empty gel. In the study, researchers fabricated devices containing multiple channels and different shapes, which increased contact surface area and toxin absorption capacity, resulting in more effective neutralization of melittins and α-toxins. This study further demonstrated the versatility of NS-gel in detoxification applications.

In another study, researchers made environment-responsive NS-gel to neutralize PFTs locally [73]. A thermosensitive and injectable NS-gel was constructed by loading RBC-NS into a 30% Pluronic F127 hydrogel. This thermosensitive NS-gel maintained fluidity and viscosity at lower temperatures ideal for local injection but quickly underwent sol–gel transition near body temperature for gelation. Besides, this hybrid system had a sustained nanosponge release behavior accompanied by good biocompatibility and biodegradability. When exposed to recombinant *Vibrio vulnificus* hemolysin (VvhA) or α-toxin, the heat-responsive NS-gel inhibited RBC lysis in vitro in a dose-dependent manner. In mice subcutaneously injected with VvhA or α-toxin, the thermosensitive NS-gel showed significant alleviation of skin lesion development in both therapeutic and prophylactic settings.

**Fig. 9.**
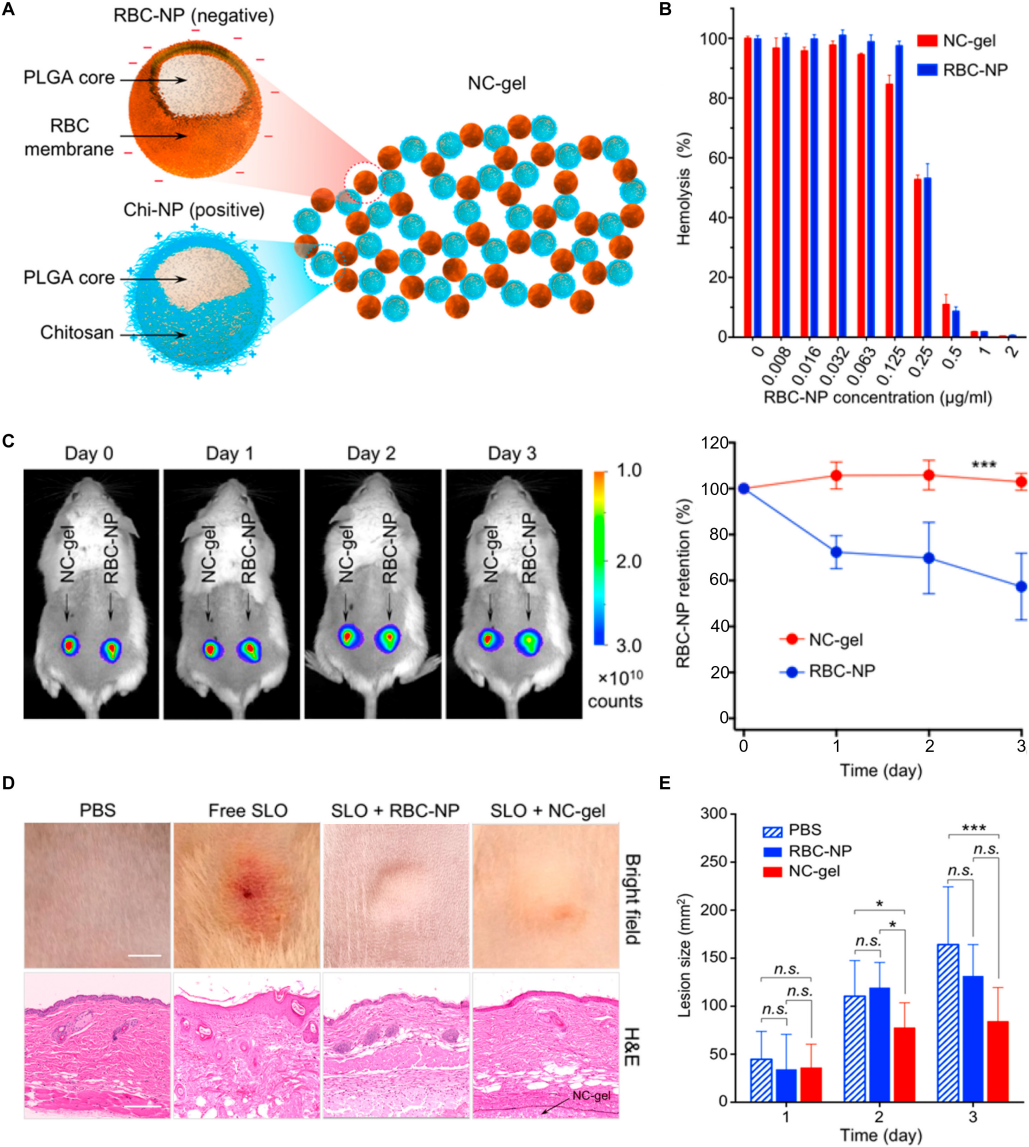
Self-assembled colloidal gel using cell membrane-coated nanosponges as building blocks. (A) Schematic illustration of a colloidal hydrogel with nanosponges as building blocks (NC-gel). The NC-gel was formulated by the electrostatic interaction between negatively charged RBC-NPs and positively charged chitosan-coated PLGA nanoparticles (Chi-NPs). (B) In vitro toxin-neutralizing using the NC-gel. In the study, 1 μg/ml of recombinant SLO was mixed with serial dilutions of NC-gel or RBC-NPs and then added to purified human RBCs. The percentage of RBC hemolysis was measured. (C) In vivo nanosponge retention within NC-gel. RBC-NPs were labeled with DiD fluorescent dye, followed by nanosponge formulation. The NC-gel was subcutaneously injected under the loose skin over the left flank of the mice. As the control group, free RBC-NPs were injected under the right flank of the same mice in the same route. The nanosponge retention was observed via fluorescence imaging (left) and quantification of detected fluorescence intensity (right) on days 0, 1, 2, and 3 after the injection (*n* = 3). Error bars represent the SD; ***P < 0.001. (D) In vivo toxin neutralization with NC-gel. In the study, CD-1 mice were subcutaneously injected with SLO, followed by the administration of PBS, free RBC-NPs, or NC-gel in the same route. After 72 h, the developed skin lesions were photographed (upper). The H&E-stained histological sections showed inflammatory infiltrate, apoptosis, necrosis, and edema in the epidermis (lower). (E) In vivo antibacterial evaluation of NC-gel protection from GAS infection. At first, 2 × 10^9^ CFU of GAS were subcutaneously injected into the backs of mice (*n* = 6), and the treatment of PBS, free RBC-NPs, or NC-gel was immediately followed in the same route. Skin lesion sizes were then monitored on days 1 to 3 after injection. The bars represent median values. *P < 0.05, ***P < 0.001. Figure adapted from [74].

Besides entrapping cellular nanosponges into gel networks, researchers also used cellular nanosponges directly as crosslinkers to assemble nanosponge–colloidal gel (denoted NC-gel) for local detoxification (Fig. 9) [74]. In this development, negatively charged RBC-NS were mixed with positively charged, chitosan-coated PLGA nanoparticles (denoted “Chi-NPs”). The 2 oppositely charged nanoparticles self-assembled and formed NC-gel. By optimizing the ratio of the 2 nanoparticles, the NC-gel showed a high viscosity at a static condition but notable shear-thinning under high shear stress, a feature ideal for local injection. The NC-gel inhibited RBC hemolysis against streptolysin-O toxin (SLO) secreted from group A *Streptococcus* (GAS) in vitro and prolonged RBC-NS retention in vivo. When mice were subcutaneously injected with SLO or GAS, local injection of NC-gel reduced the skin lesion significantly, indicating a robust toxin-neutralizing capacity of NC-gel and its therapeutic potential.

Overall, recent efforts have developed NS-gel by directly embedding nanosponges into the hydrogel or constructing nanosponge colloidal gels by applying oppositely charged nanoparticles. Both approaches highlighted the strong local detoxification effect of NS-gel systems and their promise for future antivirulence therapy.

## Conclusion and outlook

In the past few years, cell membrane-coated nanoparticles have advanced substantially, especially in the areas of understanding cellular nanosponge structure–function relationships, synthesizing effective and multifunctional nanosponge formulations, and controlling biointerfacing properties in complex biological systems. Efforts in these aspects have led to various cellular nanosponges that address medical countermeasure challenges facing traditional techniques. Major design strategies reviewed in this article include harnessing native cell membrane functions for biological neutralization, functionalizing cell membrane coatings to enhance neutralization capabilities, combining cell membranes and functional cores for multimodal neutralization, and integrating cellular nanosponges with hydrogels for localized applications. Representative nanosponge examples are selected in each design category and discussed for their structural features, formulation strategy, biointerfacing capability, and therapeutic outcome. The discussion highlights the structure–function relationship in each design category and reveals how such relationships lead to potent neutralization efficacy in complex disease settings. Examples reviewed here may inspire novel designs for future applications.

Notably, the clinical translation of cellular nanosponges still faces multiple challenges ranging from large-scale manufacturing to regulatory acceptance. Consisting of entire cellular plasma membrane, the composition of cellular nanosponges includes large amounts of membrane proteins and lipids, which are much more complex than the traditional small-molecule and biological drugs. Therefore, nanosponge manufacturing requires important quality controls involving their size, surface chemistry, membrane composition, and biological potency. Batch-to-batch consistency is of great importance in producing a large amount of cellular nanosponges toward clinical trials and commercialization. As there is no existing regulatory pathway to follow, cellular nanosponge needs to initiate its own regulatory strategy toward clinical translation, which will likely involve extensive communications among drug developers, regulatory agencies, and clinical experts. While challenges exist, cellular nanosponges are expected to continue to bring novel formulations for potent, patient-compliant, and cost-effective countermeasure therapeutics.
